# Incident Cancer Risk of Patients with Prevalent Type 2 Diabetes Mellitus in Hungary (Part 2)

**DOI:** 10.3390/cancers16132414

**Published:** 2024-06-29

**Authors:** Zsolt Abonyi-Tóth, György Rokszin, Gábor Sütő, Ibolya Fábián, Zoltán Kiss, György Jermendy, Péter Kempler, Csaba Lengyel, István Wittmann, Gergő A. Molnár

**Affiliations:** 1 RxTarget Ltd., 5000 Szolnok, Hungary; abonyi-toth.zsolt@rxtarget.hu (Z.A.-T.); rokszin.gyorgy@rxtarget.hu (G.R.); fabian.ibolya@rxtarget.hu (I.F.); 2 Department of Biostatistics, University of Veterinary Medicine, 1078 Budapest, Hungary; 3 Second Department of Medicine and Nephrology-Diabetes Centre, University of Pécs Medical School, 7624 Pécs, Hungary; suto.gabor@pte.hu (G.S.); zoltan_kiss2@merck.com (Z.K.); molnar.gergo@pte.hu (G.A.M.); 4 Department of Internal Medicine, Bajcsy-Zsilinszky Hospital, 1106 Budapest, Hungary; gyjermendy@gmail.com; 5 Department of Medicine and Oncology, Faculty of Medicine, Semmelweis University, 1089 Budapest, Hungary; kemplerpeter@gmail.com; 6 Department of Internal Medicine, University of Szeged, 6720 Szeged, Hungary; lecs@in1st.szote.u-szeged.hu

**Keywords:** diabetes mellitus (type 2), cancer, incidence, age, epidemiology

## Abstract

**Simple Summary:**

This study aimed to determine the risk and rate of development of overall and site-specific cancer in patients with type 2 diabetes mellitus compared with non-diabetic individuals. In this retrospective cohort study, excess incidence of cancer was found in patients with type 2 diabetes mellitus. While the incidence of cancer decreased in non-diabetic controls between 2015 and 2019 in most age groups and for several cancer sites, this decrease was less significant among patients with type 2 diabetes mellitus. Therefore, the results of this study demonstrate that the presence of type 2 diabetes mellitus leads to a higher incidence of cancer.

**Abstract:**

(1) Background: Among the chronic complications of type 2 diabetes mellitus, cancer has become the leading cause of death in several countries. Our objective was to determine whether prevalent type 2 diabetes mellitus is associated with a higher incidence of cancer. (2) Methods: This study comprised a nationwide analysis conducted in Hungary. The study population was divided into two groups: a type 2 diabetes mellitus group vs. a non-diabetic group. The primary outcome was the risk related to overall cancer incidence; a key secondary outcome was the overall incidence of cancer in distinct study years; and a further outcome was the annual percent changes. (3) Results: The odds ratio related to the overall incidence of cancer was 2.50 (95% confidence interval: 2.46–2.55, *p* < 0.0001) in patients with diabetes as related to non-diabetic controls. The odds ratio was higher in males than in females [OR_males_: 2.76 (2.70–2.82) vs. OR_females_: 2.27 (2.22–2.33), *p* < 0.05 for male-to-female comparison]. The annual cancer incidence rate declined in non-diabetic controls, but not in patients with diabetes [−1.79% (−2.07–−1.52%), *p* < 0.0001] vs. −0.50% (−1.12–+0.10%), *p* = 0.0991]. Several types of cancer showed a decreasing tendency in non-diabetic controls, but not in patients with type 2 diabetes. (4) Conclusions: Type 2 diabetes is associated with a higher risk of cancer. While the cancer incidence decreased for non-diabetic individuals with time, it remained unchanged in patients with T2DM.

## 1. Introduction

Type 2 diabetes mellitus (T2DM) is a disease with a worldwide high and increasing prevalence [1]. In recent decades, cardiovascular and renal risk have become the focus of treatment of T2DM [2]. However, improvements in pharmacological and non-pharmacological therapeutic approaches have ameliorated cardiovascular disease (CVD) and renal outcomes [3]; therefore, CVD-related mortality decreases faster than the change in cancer-related mortality [4]. In fact, research has shown that “Cancer is becoming the leading cause of death in diabetes” [5]. For example, in Australia, between 1997 and 2010, CVD-related mortality decreased while cancer-related mortality increased [6]. A study from Hong Kong found that for those in the age group between 45–74 years, cancer was the leading cause of mortality in patients with diabetes [7]. A Swedish national observation study showed that CVD-related mortality decreased in patients with T2DM and in controls between 1998 and 2012, whereas cancer-related mortality increased. The study suggests that cancer would expectedly be the leading contributor to mortality in patients with T2DM by 2030 and in the control group by 2040 [8]. Researchers from England found that for those in the age groups between 75–85 and 85+ years, the risk of cancer increased by 1.2% and 1.6% annually [9]. Data from Scotland verified that mortality due to CVD has decreased, while mortality related to cancer has become the leading cause of mortality in diabetes, reaching 28% by 2018 [10]. Data from the Global Burden of Disease show that cancer is the second most common cause of mortality worldwide, and, in fact, in high-income countries, cancer is the most frequent cause [11]. Thus, research related to diabetes and cancer is becoming an important area of interest.

As the majority of previous studies either selected one particular type of cancer or focused on the effect of age and gender, providing data on temporal changes separately, the present study aimed to obtain robust, nationwide data on the risk of cancer development in patients with T2DM. Moreover, we also aimed to report on the effect of age and gender and analyze temporal trends in order to present the results on these parameters for 20+ cancer sites in a single article. Furthermore, we aimed to provide a brief overview of the pre-existing data of the literature. Thus, the present study is not merely a replication of previous findings.

## 2. Materials and Methods

This study was carried out in accordance with the Declaration of Helsinki [12]. The study protocol was approved by the Scientific and Research Ethics Committee of the Medical Research Council of Hungary (BMEÜ/325-1/2022/EKU). During the preparation of the manuscript, the Strengthening the Reporting of Observational Studies in Epidemiology (STROBE) guideline was followed. 

The study population was also investigated according to decades of age. Furthermore, subgroup comparisons of those aged 18–59 and 60+ years were performed. The study population cut-off was 60 years of age for two reasons: (i) this is the cut-off age suggested by the WHO to be used for the definition of the elderly population [13]; (ii) the 60+ age subgroup comprises approximately 25% of the total population and nearly 33% of the general adult population [14], providing sufficient data for analysis. 

### Data Sources

All data—consisting of data on patients with T2DM, the background population, and cancer data related to cases or controls—were obtained from the same database, i.e., the National Health Insurance Fund (NHIF) database. The official data of the Central Statistical Office (CSO) were only used to obtain the total Hungarian population number. By subtracting the number of patients from the total population count, we determined the number of non-diabetic controls. By subtracting the number of cases of cancer from the total population count, we determined the number of patients at risk of developing cancer. Based upon the ICD-10 codes, the NHIF data indicate whether a person did or did not have diabetes at a given time and whether the person suffered from cancer. However, due to data protection rules, we did not receive any person-level data; only data of selected groups and subgroups were obtained. Furthermore, the NHIF database provided only aggregated data for subgroups with ≥10 cases and the results of the statistical models.

Cancer-related cases were identified according to previously applied methods [15,16] and T2DM cases were identified similarly to those described in our previous articles [17,18,19]. Briefly, ICD-10 codes were used to identify patients with diabetes out of the patient pool. We excluded patients with gestational diabetes and polycystic ovary syndrome (PCOS). In the next step, we identified patients with type 1 diabetes mellitus based on a hierarchical definition and excluded them from the pool of patients with diabetes mellitus. The full details of the data sources and study process are provided in the Supplementary Materials of this paper and the previously published first part of this study (part 1) [20].

**Figure 1:**
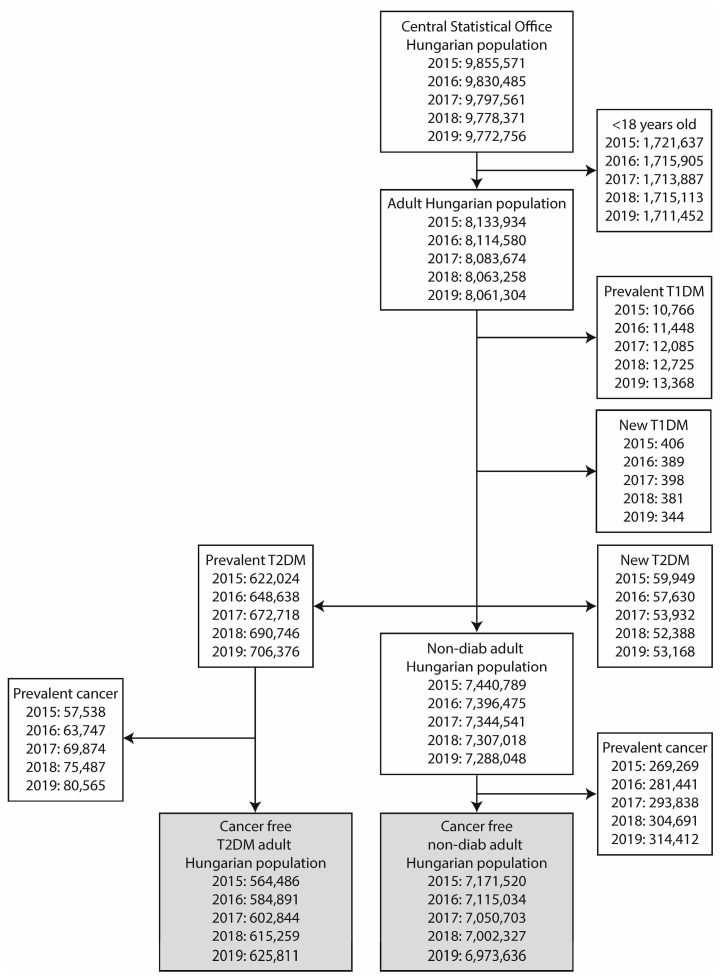
Flowchart depicting case numbers at each step of the study. The two major groups compared (prevalent T2DM cases and non-diabetic controls) are highlighted with bold style.

Cases with an age of less than 18 years were excluded from this study. Similarly, cases with type 1 diabetes mellitus, and, subsequently, cases with incident T2DM (newly diagnosed cases with T2DM, diagnosed in the index calendar year, i.e., between 1 January and 31 December of the given year) were excluded from the data of the adult population. The remaining population was divided into groups of cases with prevalent T2DM and non-diabetic controls (Figure 1).

The analysis of the incidence of cancer was carried out using R (MASS package, version 4.0.4). We used binomial logistic regression where the population in each year included people without cancer on the 1st of January, who had T2DM already or did not develop T2DM in one year. Prevalent T2DM patients and non-diabetic subjects who did not develop T2DM during the calendar year were considered. We analyzed new T2DM cases in our previously published article on this study (part 1). The dependent variable was the incidence of cancer during the calendar year, while the independent variables were taken to be the presence of T2DM, age group, gender, and the interactions of these parameters. 

The odds ratio (OR) and annual percent change (APC) in the incidence rate were calculated using bootstrap methods with one billion repetitions. The use of bootstrap methods enabled the calculation of the 95% confidence intervals related to the main statistics (OR or APC). The statistical analysis also considered interactions. We estimated the risk of cancer development for all unique age–group + gender–group combinations. The marginal estimates for age groups, genders, and the total population were calculated from these estimates using weighted averages, employing the average number of baseline populations of the subgroups during the study period as weights. The findings related to the OR in different age groups were strengthened using a further statistic, i.e., Fisher’s test.

## 3. Results

### 3.1. Data on Total Cancer Incidence with Trends

**Figure 2:**
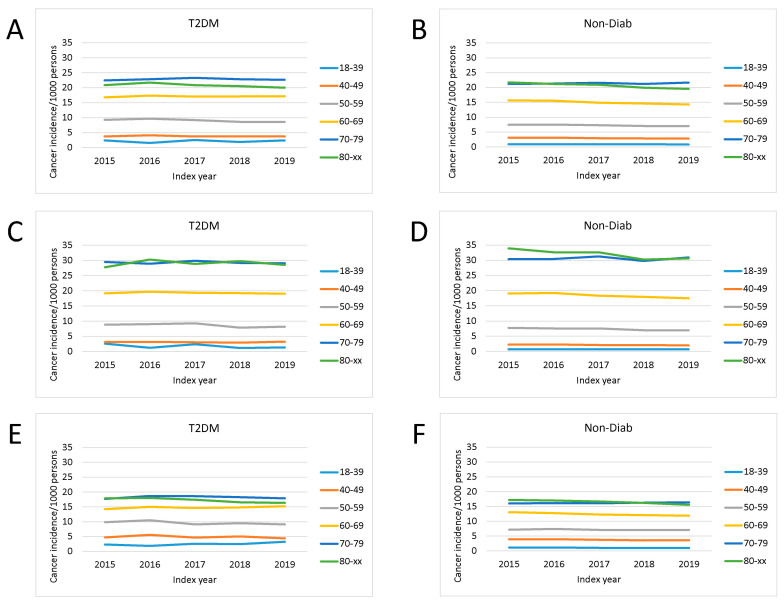
Change in incidence of cancer cases per 1000 prevalent T2DM cases (**A**,**C**,**E**) and in nondiabetic controls (**B**,**D**,**F**) in the time interval of 2015–2019 in distinct age groups. (**A**,**B**) total population, (**C**,**D**) male patients, (**E**,**F**) female patients. Crude incidence data are shown.

**Figure 3:**
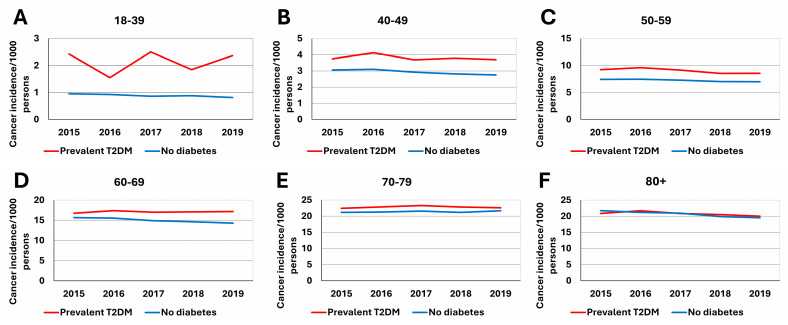
Crude cancer incidence rates per 1000 prevalent T2DM cases (red lines) or per 1000 controls (blue lines) in different age groups. Panels are: (**A**) 18–39 years, (**B**) 40–49 years (**C**) 50–59 years, (**D**) 60–69 years, (**E**) 70–79 years, (**F**) 80–89 years. Please note that incidence data are shown in different panels on different scales on the *y* axis, to underline the difference between the crude incidence of cancer in distinct age groups.

The total cancer incidence in patients with type 2 diabetes mellitus and in controls was investigated in the period from 2015 to 2019 in different age groups. The crude cancer incidence was highest in the 80+ and 70–79 age groups (Figure 2 and Figure 3). For example, absolute incidence values around 3/1000 persons were found in the 40–49 age group in the Non-Diab group, while values around 4/1000 persons were found in the same age group in the T2DM group. In the 70–79 age group, the incidence of cancer varied in the range of 20–25/1000 persons, with the T2DM group being slightly higher than the Non-Diab group. In the 80+ age group, the crude incidence was approximately the same in the two groups that were compared, and slightly declined between 2015 and 2019. The same result was observed in analyses of male and female patients separately; however, overall cancer incidence was higher in males than in females in the 60–69, 70–79, and 80+ age groups (e.g., around 30/1000 persons in the elderly males with T2DM, while the range was 15–20/1000 persons in females with T2DM, as shown in Figure 2).

The crude cancer incidence was markedly different in patients with T2DM compared with the Non-Diab group, and temporal trends were also different in the two groups (Figure 3).

### 3.2. Age Group Distribution of Cases with Cancer

**Figure 4:**
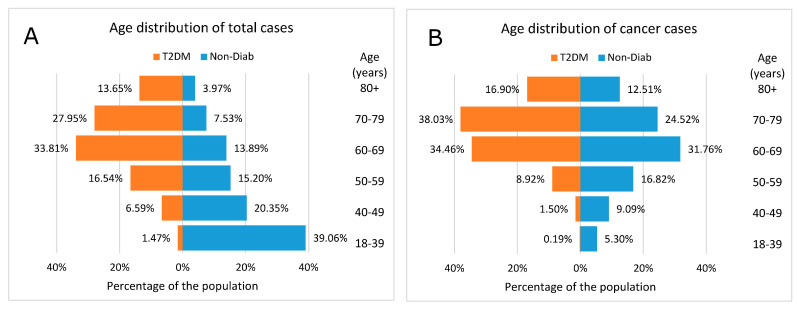
Age distribution of (**A**) total cases and (**B**) incidental cases of cancer in patients with type 2 diabetes mellitus and in nondiabetic controls. In panel (**A**), the total Hungarian population was divided to cases with and without T2DM, while in panel (**B**) cases with incident cancer only were divided to a group with or without T2DM.

A comparison of the age distribution of the total cases (i.e., the total Hungarian population was divided into cases with and without T2DM) showed that the 60+ age groups dominated the T2DM group. Conversely, in the Non-Diab group, the <60 age groups accounted for nearly 75% of all cases (Figure 4A). 

The age pyramid of cases with incident cancer (with or without T2DM) shows that the age distribution was markedly different in the two groups, with more young patients in the Non-Diab group and more elderly patients in the T2DM group (Figure 4B).

### 3.3. Cancer Risk and Temporal Changes in the Incidence of Cancer 

**Figure 5:**
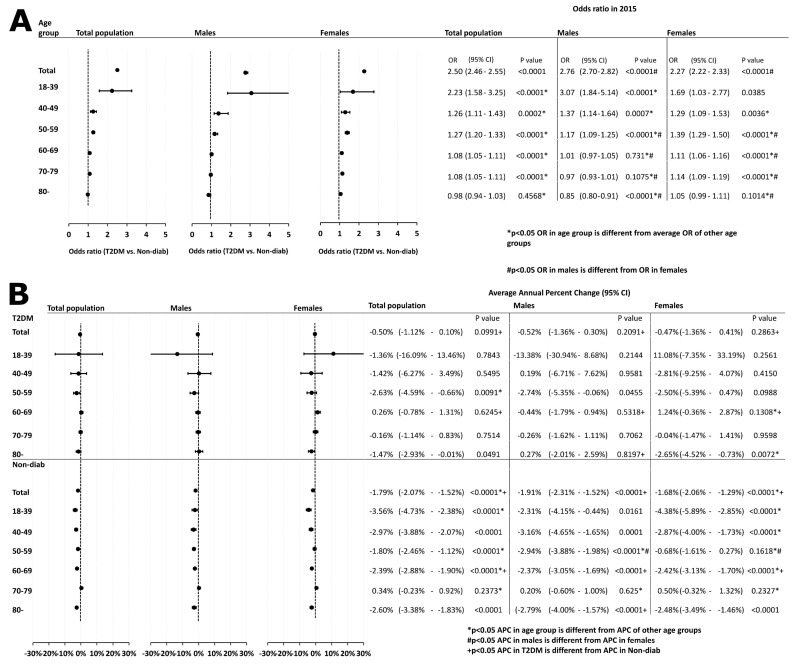
Data related to incidental cancer development in different age groups, irrespective of location and histological type. (**A**) odds ratio of cases with T2DM vs. controls is shown for the total population, both males and females, respectively. (**B**) average annual percent changes in total cancer incidence in cases with T2DM and controls. Also here, data of the total population, as well as male and female cases are shown.

Furthermore, we analyzed the odds of developing cancer and found that patients with T2DM had a significantly higher cancer risk [OR: 2.50 (2.46–2.55)] than those in the Non-Diab group. When analyzing the risk in different age groups, we found that an elevated OR was present in all age groups [18–39 years, OR: 2.23 (1.58–3.25), *p* < 0.0001; 40–49 years, OR: 1.26 (1.11–1.43), *p* < 0.0001; 50–59 years, OR: 1.27 (1.20–1.33), *p* = 0.0002; 60–69 years, 1.08 (1.05–1.11), *p* < 0.0001; 70–79 years, OR: 1.08 (1.05–1.11), *p* < 0.0001], except for the 80+ group [OR: 0.98 (0.94–1.03), *p* = 0.4568]. For sensitivity analysis, we used Fisher’s exact test to check the OR of cancer in the 2015 cohort in the different age groups, and we received identical results (Supplementary Materials, Table S1). Moreover, the overall cancer risk of male patients was higher than that of female patients [OR: 2.76 (2.70–2.82) vs. OR: 2.27 (2.22–2.33), *p* < 0.05]. The OR was higher in females than in males in the 50+ age groups [50–59 years: OR_females_: 1.39 (1.29–1.50) vs. OR_males_: 1.17 (1.09–1.25), *p* < 0.05; 60–69 years: OR_females_: 1.11 (1.06–1.16) vs. OR_males_: 1.01 (0.97–1.05), *p* < 0.05; 70–79 years: OR_females_: 1.14 (1.09–1.19) vs. OR_males_: 0.97 (0.93–1.01), *p* < 0.05; 80+: OR_females_: 1.05 (0.99–1.11) vs. OR_males_: 0.85 (0.80–0.91), *p* < 0.05], but was not different in the group of 40–49-year-olds [OR_females_: 1.29 (1.09–1.53) vs. OR_males_: 1.37 (1.14–1.64), *p* > 0.05] (Figure 5A).

Next, we studied the temporal change in overall cancer in the total population and in different age groups. In the total population, no significant trend could be observed in the T2DM group [APC: −0.50% (−1.12–0.10%), *p* = 0.0991], while a decreasing trend was found in the Non-Diab group [APC: −1.79% (−2.07–−1.52%), *p* < 0.0001]. When analyzing different age groups separately, in the T2DM group, there was a decreasing trend in the 50–59 age group [APC: −2.63% (−4.59–−0.66%), *p* = 0.0091] and the 80+ age group [APC: −1.47 (−2.93–−0.01%), *p* = 0.049] only. In the Non-Diab group, there was a decreasing trend in all age groups [18–39 years: APC: −3.56% (−4.73–−2.38%), *p* < 0.0001; 40–49 years: APC: −2.97% (−3.88–−2.07%), *p* < 0.0001; 50–59 years: APC: −1.80% (−2.46–−1.12%), *p* < 0.0001; 60–69 years: APC: −2.39% (−2.88–−1.90%), *p* < 0.0001; 80+: APC: −2.60% (−3.38–−1.83%), *p* < 0.001], except for the 70–79 age group [APC: 0.34% (−0.23–0.92%), *p* = 0.2373]. No difference could be detected between males and females in the APC, nor in patients with T2DM [APC_males_: −0.52% (−1.36–0.30%) vs. APC _females_: −0.47% (−1.36–0.41%), *p* > 0.05] nor in the Non-Diab group [APC_males_: −1.91% (−2.31–−1.52%) vs. APC _females_: −1.68% (−2.06–1.29%), *p* > 0.05]. Overall, among patients with T2DM, there was no difference in the APC between males and females in any age group (*p* > 0.05 for all), whereas in the Non-Diab group, the APC showed a more marked decrease in the 50–59-year-old males than in females [APC_males_: −2.94% (−3.88–−1.98%) vs. APC _females_: −0.68% (−1.61–0.27%), *p* < 0.05] (Figure 5B). 

### 3.4. Data on Site-Specific Incidence of Cancer 

#### 3.4.1. Site-Specific Distribution of Cancer Types in the T2DM vs. Non-Diab Groups

**Figure 6:**
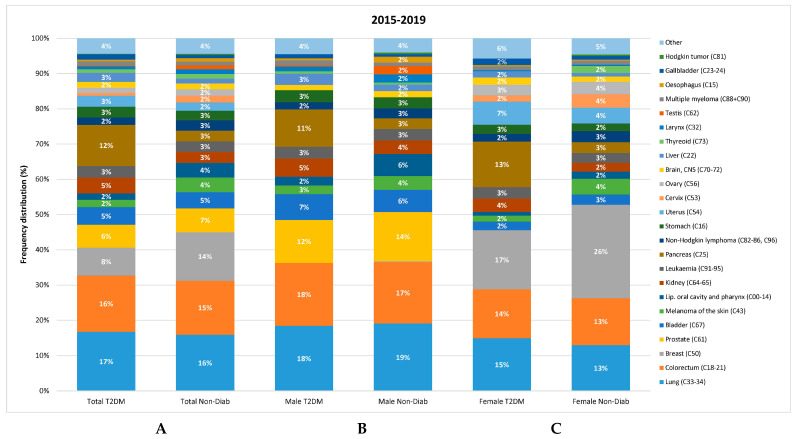
Cumulative frequency (crude, non-adjusted data) of different cancer types in non-diabetic controls and in cases with Type 2 diabetes. (**A**) total data, (**B**) cancer site distribution in males, (**C**) cancer site distribution in female patients.

In both groups, the four most common cancer types (lung, colorectal, breast, and prostate) accounted for approximately 50% of cancer cases (Figure 6).

The relative distribution of individual cancer sites was slightly different in the T2DM and Non-Diab groups: colorectal, prostate, bladder, kidney, pancreas, stomach, uterus corpus, and liver cancers accounted for a larger proportion of all cancer cases in the T2DM group than in the Non-Diab group. On the contrary, lung, breast, melanoma, oral, leukemia, non-Hodgkin lymphoma, ovary, brain, thyroid, larynx, and testis cancers accounted for a larger proportion of cancer cases in the Non-Diab group than the T2DM group (Figure 6). 

**Figure 7:**
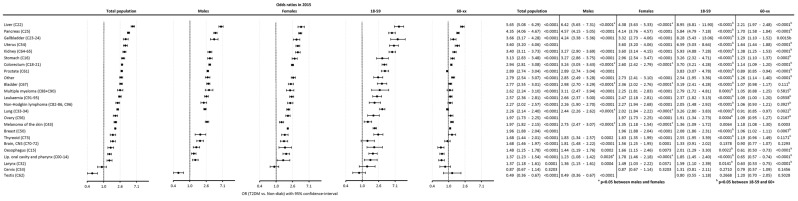
Odds ratio of development of cancer in T2DM cases compared to controls. Detailed data for individual cancer sites are shown. The panel shows data for the total population, for a male-to-female comparison as well as comparison of 18–59 vs. 60+ age groups.

The odds of developing new cancer were highest for those with liver cancer [OR: 5.65 (5.08–6.29)], followed by pancreas [OR: 4.35 (4.06–4.67)], gallbladder [OR: 3.66 (3.17–4.28)], uterus [OR: 3.60 (3.20–4.06)], kidney [OR: 3.40 (3.11–3.73)], and other types of cancer. On the contrary, the odds were lower in the T2DM group for testis cancer compared with the controls [OR: 0.49 (0.36–0.67)] (Figure 7).

When comparing males to females, the risk in males was higher for liver [OR_males_: 6.42 (5.65–7.31) vs. OR_females_: 4.38 (3.63–5.33)], colorectal [OR_males_: 3.24 (3.05–3.43) vs. OR_females_: 2.60 (2.42–2.79)], bladder [OR_males_: 2.98 (2.70–3.29) vs. OR_females_: 2.36 (2.02–2.76)], lung [OR_males_: 2.44 (2.26–2.62) vs. OR_females_: 2.02 (1.84–2.22)], and melanoma cancers [OR_males_: 2.75 (2.47–3.07) vs. OR_females_: 1.35 (1.18–1.54)], while it was higher in females than in males for cancers of the lip, oral cavity, and pharynx combined [OR_males_: 1.23 (1.08–1.42) vs. OR_females_: 1.78 (1.46–2.18)] (Figure 7).

In younger patients (18–59 years), T2DM was associated with significantly higher odds of developing cancer for most cancer types except for brain [OR: 1.33 (0.91–2.02), *p* = 0.1378], cervix [OR: 1.31 (0.81–2.11), *p* = 0.2710], and testis cancers [OR: 0.80 (0.55–1.18), *p* = 0.2668]. In the older age group (60+), T2DM was associated with higher odds for several cancer sites, except for bladder, myeloma, leukemia, non-Hodgkin lymphoma, ovary, thyroid, brain, cervix, and testis cancers (*p* > 0.05 for the latter groups) (Figure 7).

When comparing the 18–59 and the 60+ age groups, the excess risk of diabetic patients in the 18–59 age group was higher for nearly all cancer types (including colorectal, lung, prostate, and breast cancers) (Figure 7).

#### 3.4.2. Temporal Trends of Site-Specific Cancer Incidence

**Figure 8:**
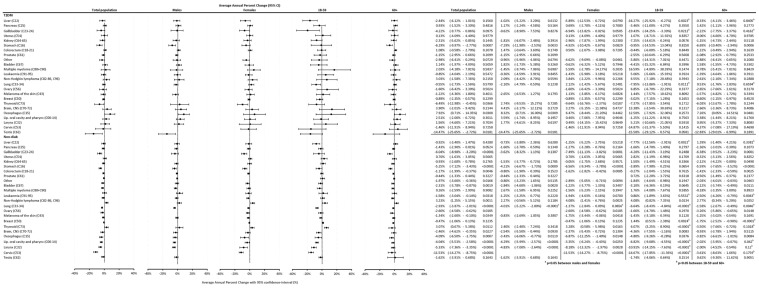
Average annual percent changes in incidence for individual cancer types in cases with T2DM and in controls. Detailed data for individual cancer sites. Data for the total population, for a male-to-female comparison as well as comparison of 18–59 vs. 60+ age groups are shown.

Analyzing temporal trends using the APC for the total population in the T2DM group, we found a significant increasing tendency for esophagus cancer [APC: 7.92% (0.71–14.35%), *p* = 0.0300], and a significant decreasing tendency for stomach [APC: −6.29% (−9.97–−2.77%), *p* = 0.0007], thyroid [APC: −6.49% (−12.98–−0.45%), *p* = 0.0366], and testis [APC: −14.47% (−25.65–−2.72%), *p* = 0.0181] cancers. As for the Non-Diab group, we found an increasing trend for non-Hodgkin lymphoma [APC: 3.23% (1.25–5.15%), *p* = 0.0011] and thyroid [APC: 3.07% (0.67–5.38%), *p* = 0.0112] cancers, and a decreasing trend for gallbladder [APC: −6.04% (−8.98–−3.20%), *p* < 0.0001], stomach [APC: −5.25% (−7.12–−3.43%), *p* < 0.0001], colorectal [APC: −1.17% (−1.99–−0.37%), *p* = 0.0046], lung [APC: −2.93% (−3.87–−2.01%), *p* < 0.0001], ovarian [APC: −2.60% (−4.58–−0.62%), *p* = 0.0105], brain [APC: −2.46% (−4.62–−0.35%), *p* = 0.0227], esophagus [APC: −4.09% (−6.50–−1.75%), *p* = 0.0007], pharynx [APC: −4.04% (−5.53–−2.58%), *p* < 0.0001], larynx [APC: −5.33% (−7.36–−3.35%), *p* < 0.0001], and cervix [APC: −11.53% (−14.27–−8.75%), *p* < 0.0001] cancers (Figure 8).

Comparing males to females, we only found a difference in the Non-Diab group and only for lung cancer [APC_males_: −4.01% (−5.22–−2.80%) vs. APC_females_: −1.37% (−2.08–0.09%), *p* < 0.05], but not in the T2DM group and not for other cancer sites (Figure 8).

When comparing the 18–59 to the 60+ age groups, in the T2DM group, a decreasing tendency was more expressed in the 18–59 age group for liver, gallbladder, and lung cancers, but not for other types of cancer. In the Non-Diab group, the decreasing tendency was more expressed in the 18–59 age group for liver, lung, pharynx, larynx, and cervix cancers, while it was more expressed in the 60+ age group for leukemia. In the case of breast cancer, the incidence significantly increased in the younger and significantly decreased in the older non-diabetic patients. For thyroid cancer, the incidence increased significantly in the younger but did not change in the older non-diabetic patients (Figure 8). 

### 3.5. Relative Age Distribution

**Figure 9:**
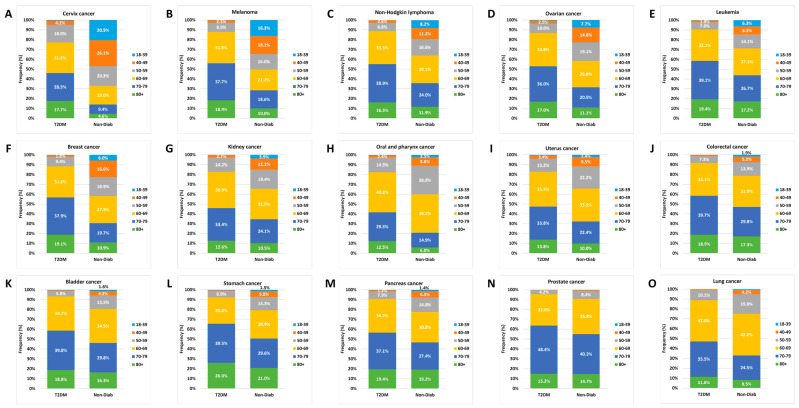
Age distribution of site-specific incident cancer types in non-diabetic controls and in patients with type 2 diabetes mellitus. Cancer sites are ordered according to the relative frequency of the 18–39-year-old group in non-diabetic controls.

We also analyzed the percentages of the age groups within the cancer cases in the two groups and found striking differences, especially for cervix, melanoma, non-Hodgkin lymphoma, ovarian, leukemia, and breast cancer types, where the proportion of younger age groups was higher in the controls than in the T2DM cases. On the other hand, patients aged 70+ dominated the T2DM group for all individual cancer sites, while the relative proportion of these patients was lower in the Non-Diab group (Figure 9).

## 4. Discussion

The present study found that the incidence of cancer was generally higher in males than in females, both in patients with T2DM and in the Non-Diab group. Cancer affected different age groups differently in patients with T2DM and in the controls. In Non-Diab cases, the incidence of cancer decreased overall and in several age groups. On the other hand, there was no decrease in the incidence of cancer in the T2DM group. Moreover, the site-specific distribution of cancer types was different, e.g., the relative contribution of kidney, pancreas, and liver cancers was higher in patients with T2DM, whereas the relative contribution of breast, oropharyngeal, and testis cancers was higher in the Non-Diab groups. When analyzing the OR of cancer of individual sites in the T2DM vs. the Non-Diab groups, we found that the odds were highest for liver, pancreas, gallbladder, uterus, kidney, and colorectal cancers. For most cancer types, the OR of patients with T2DM developing cancer was higher in the <60 age group vs. the 60+ age group. In the Non-Diab group, the annual incidence declined for gallbladder, stomach, colorectal, lung, brain, esophagus, pharynx, larynx, and cervix cancers, while in the T2DM group, only the incidence of stomach, thyroid, and testis cancers decreased. In the Non-Diab group, the annual incidence increased for non-Hodgkin lymphoma and thyroid cancers, while in the Non-Diab group, it increased only for esophagus cancer.

Regarding the association between overall cancer risk and T2DM, we verified the positive association suggested in the literature. Concerning the overall incidence of cancer, the hazard ratio (HR) was 1.21 (1.16–1.26) in one analysis [21], whereas the SIR in another study was 1.22 for male and 1.737 for female patients with T2DM [22]. An umbrella review of meta-analyses found a positive association with T2DM for breast, liver, endometrium, and colorectal cancers [23]. In a Swedish national study, overall cancer risk had a slight positive association with T2DM [HR: 1.10 (1.09–1.12)] [8]. Italian authors found an incidence rate ratio (IRR) of 1.22 (1.15–1.29) in patients with DM, with the strongest association with liver, pancreas, colorectal, urinary bladder, and uterus corpus cancers [24], while in our study, the strongest associations were found for liver, pancreas, gallbladder, uterus, and kidney cancers. 

Data from the literature are quite heterogeneous, and different measures (IRR, standardized incidence rate (SIR), relative risk (RR), HR, and OR) have been used in different studies and for different study designs. At the same time, data can be markedly different for different cancer sites (Table 1). 

In general, the risk of patients with T2DM developing cancer is higher in the present study compared with the literature; this result might have multiple explanations. Obesity is a well-known risk factor for both T2DM and cancer and has been found to act as a confounder in epidemiological studies analyzing the association between T2DM and cancer [39]. Several of the above-mentioned studies (e.g., [3,10,12,32,37,40,41,42,43,44,45,46,47]) used adjustment to anthropometric markers including markers for obesity, such as body mass index (BMI), which could have severely altered their results. In the present study, we did not perform any adjustment for BMI or other anthropometric parameters, as BMI data were not available from the NHIF database. This factor might have contributed to the higher OR values compared with some studies from the literature. However, obesity is a hallmark of T2DM for the majority of the patients in this study; thus, correcting for BMI in the analysis could produce an inaccurate estimation of the real excess risk caused by T2DM. Moreover, data on lifestyle factors such as exercise, smoking, and eating habits were not available from the NHIF database. We believe that correction in terms of these factors would have “overcorrected” the statistical connection between T2DM and cancer.

Moreover, demographic and ethnic markers of the Hungarian population could also have influenced the results compared with data taken from other geographic areas.

With regard to temporal trends, our data indicate a decreasing cancer incidence trend in the Non-Diab group and nearly all age groups. However, in the T2DM group, no beneficial trend could be observed, except for the 50–59 and 80+ age subgroups. Global data on metabolic-associated cancer show a worldwide overall increasing trend [AAPC: 0.74 (0.71–0.76)] [48]. The Global Burden of Disease study also placed Hungary in the category with a −0.9–0% annual percent change in cancer incidence; however, the results of the present study demonstrated APC values of −1.78 and −0.5 for the Non-Diab and T2DM groups, respectively [11].

The strengths of our study include its nationwide approach with an almost 100% coverage of the Hungarian population, enabling robust results. The selected database also facilitated simultaneous analysis of the effect of age groups, gender, individual cancer sites, and temporal changes.

The limitations of our study include the lack of anthropometric (such as weight, BMI, waist circumference, weight-to-hip ratio—that may influence the statistical relation, as obesity is a known risk factor for several typers of cancer), clinical, and laboratory data (such as HbA_1c_), as well as the retrospective nature of the research. Our results do not provide any data about the risk of cancer in people with type 1 DM or adolescents. Moreover, the major difference in the age distribution of the two groups was a significant impediment, which could only be addressed by employing statistical tools. 

## 5. Conclusions

The presence of T2DM indicates a 150% increase in the risk of developing cancer. The results of this study demonstrate that the distribution of cancer site and age in the T2DM and Non-Diab groups is different, and temporal trends are also less favorable in the T2DM group. Altogether, the results of this study underline the importance of cancer surveillance in patients with T2DM, who should be regarded as high-risk individuals.

## Figures and Tables

**Table 1:** Site-specific cancer risk in the literature and in our study.

Cancer Type	Data from the Literature				Our Data
Lung cancer	OR: 1.16 (1.03–1.31) [25]	HR: 1.21 (1.07–1.38) [21]			OR: 2.26 (2.14–2.40)
Colorectal cancer	Meta HR: 1.21 (1.06–1.38) [26]	RR: 2.05 (1.79–2.34) [26]			OR: 2.94 (2.81–3.08)
Breast cancer	Meta RR: 1.20 (1.12–1.28)	Meta RR: 1.72 (1.47–2.00)	RR: 1.25 (1.20–1.29) [27]	OR: 0.55 (0.45–0.66) [25]	OR: 1.96 (1.88–2.04)
	HR: 1.30 (1.20–1.41) [21]				
Prostate cancer	OR: 1.14 (0.93–1.40) [25]	RR: 0.90 (0.80–1.02)			OR: 2.89 (2.74–3.04)
Bladder cancer	HR: 1.17 (1.05–1.30) [28]	HR: 1.04 (0.85–1.28) [21]	HR: 0.84 (0.63–1.13) [29]		OR: 2.77 (2.55–3.01)
Melanoma	RR: 0.93 (0.64–1.36) [22]				OR: 1.97 (1.82–2.15)
Oropharyngeal cancer	OR: 0.89 (0.76–1.04) [25]				OR: 1.37 (1.23–1.54)
Kidney cancer	OR: 1.7 (1.3–2.1) [30]	HR: 1.36 (1.05–1.76) [21]			OR: 3.40 (3.11–3.73)
Pancreas cancer	Rev. RR: 0.83–6.90 * [31]	Rev. RR: 1.73–6.08 ** [31]	HR: 2.13 (1.76–2.58) [21]	OR: 1.40 (1.07–1.84) [32]	OR: 4.35 (4.06–4.67)
Stomach cancer	OR: 1.19 (0.97–1.46) [25]				OR: 3.13 (2.83–3.48)
Endometrial cancer	Meta: RR: 2.74 (1.87–4.00) [27]	HR: 1.85 (1.36–2.50) [27]	HR: 1.79 (1.51–2.13) [21]	rev. RR: 2.10 (1.75–2.53) [33]	OR: 3.60 (3.20–4.00)
	Rev. RR: 1.81 (1.38–2.37) [33]	Rev. HR: 1.81 (1.37–2.41) [33]	IRR: 1.84 (1.33–2.56) [24]	SIR: 1.75 (1.67–1.83) [34]	
	OR: 1.38 (1.07–1.80) [25]				
Cervix cancer	SIR: 1.18 (1.06–1.32) [34]	SIR: 1.18 (1.06–1.32) [34]	OR: 1.24 (1.19–1.29) [35] ^a^	OR: 1.00 (0.95–1.05) [35] ^b^	OR: 0.87 (0.67–1.14)
Ovarian cancer	Meta: OR: 1.17 (1.02–1.33) [27]	HR: 0.84 (0.61–1.15) [21]	RR: 1.05 (0.75–1.46) [33]	OR: 0.83 (0.61–1.13) [25]	OR: 1.97 (1.73–2.25)
Liver cancer	OR: 1.81 (1.66–1.97)	HR: 3.73 (2.50–5.56)	OR: 2.19 (1.76–2.72)		OR: 5.65 (5.08–6.29)
Thyroid cancer	OR: 0.70 (0.46–1.06) [36]	OR: 0.40 (0.20–0.81) [36]	OR: 0.36 (0.23–0.58) [25]	HR: 1.63 (1.14–2.34) [21]	OR: 1.68 (1.44–2.01)
Non-Hodgkin lymphoma	RR: 1.21 (0.99–1.48) [22]				OR: 2.27 (2.02–2.57)
Myeloma	RR: 1.27 (0.98–1.66) [22]	HR: 1.14 (0.80–1.65) [21]			OR: 2.62 (2.24–3.10)
Leukemia	RR: 0.88 (0.71–1.10) [22]	HR: 1.07 (0.77–1.49) [21]			OR: 2.57 (2.36–2.81)
Esophagus cancer	Meta: RR: 1.28 (1.12–1.47) [37]	HR: 1.96 (1.36–2.82) [21]	OR: 0.50 (0.35–0.71) [25]		OR: 1.48 (1.25–1.78)
Gallbladder cancer	HR: 1.47 (0.91–2.39) [21]	RR: 1.56 (1.36–1.79) [38]			OR: 3.66 (3.17–4.29)
Testis cancer	SIR: 0.94 (0.75–1.17) [34]				OR: 0.49 (0.36–0.67)

HR: hazard ratio; OR: odds ratio; RR: relative risk; SIR: standardized incidence rate; Meta: result from a meta-analysis; Rev.: data reported in a review; *: case–control studies; **: cohort studies; ^a^: univariate analysis; ^b^: multivariate analysis.

## Data Availability

Data generated during this study are available upon reasonable request from the corresponding author. Individual, patient-level data are not available due to data protection rules of the NHIF.

## Supplementary Materials

The following supporting information can be downloaded at: www.mdpi.com/article/10.3390/cancers16132414/s1. Table S1: Odds ratio of cancer incidence in cases with T2DM as compared to non-Diab individuals. Overall data as well as age-group specific data are shown. Odds ratio and 95% confidence data are provided. Data were analyzed using Fisher’s exact test.
