# Supplementary material for: Incident Cancer Risk of Patients with Prevalent Type 2 Diabetes Mellitus in Hungary (Part 2)
Source: Cancers (Basel). 2024 Jun 29;16(13):2414. doi: 10.3390/cancers16132414 (PMC11240453; doi:10.3390/cancers16132414)
Supplement: Supplementary file 1 [file cancers-16-02414-s001.zip › cancers-3039591-supplementary.pdf]

Supplementary Table S1. Odds ratio of cancer incidence in cases with T2DM as compared to non-Diab individuals. Overall data as well as age-group specific data are shown. Odds ratio and 95% confidence data are provided. Data were analyzed using Fisher's exact test.

	Odds ratio	95% confidence interval	*p* value (Fisher's exact test)
Total:	2.48	(2.42–2.53)	<0.0001
Age groups:			
18–39:	2.55	(1.53–3.99)	0.0003
40–49:	1.23	(1.03–1.45)	0.0203
50–59:	1.24	(1.16–1.33)	<0.0001
60–69:	1.07	(1.03–1.11)	0.0006
70–79:	1.06	(1.02–1.10)	0.0028
80+:	0.96	(0.91–1.01)	0.1504

SUPPLEMENTARY METHODS

Methods of identification of cancer and T2DM cases

For the cancer cases, we considered ICD Codes starting with C, except for C44 and C77-80. We used Ferlay's definition to group the ICD codes to determine the cancer sites17. In the case of multiple cancers, we considered only the sites having the majority of ICD codes and ignored all the others. The supposed date of diagnosis was the occurrence of the first ICD code in in- or outpatient records for patients who had at least two records with different date of the same cancer. Data were available between 1st January 2009 and 31st December 2022.

For T2DM cases, we considered ICD-10 E10-14 codes in in- and outpatient and drug refill records. We included those patients who had ICD codes followed by at least 30 and at most 365 days by another ICD code in the records to avoid supposed but not proved cases. The date of diagnosis was the date of the first ICD code meeting these criteria. We also included patients who died in 60 days after the occurrence of the diabetes ICD code. Finally, we also included patients having at least two refills with ATC A10 independently of ICD codes. We did not consider diabetic patients who had any polycystic ovary syndrome (ICD E28.2) or gestational DM (O24.4) code. Data was available between 1st January 2013 and 31st December 2020.

Because of possible coding errors we used a hierarchical definition to separate T1DM and T2DM cases. We considered patients having T1DM if (main rule):

1. they had E10 code, the number of E10 codes were equal to or more than the number of E11 codes, they had insulin refills and they had no OAD (oral antidiabetics) refill over 180 days of the first insulin refill.
2. (some irregular OAD): they had E10 code, the number of E10 codes were equal to or more than the number of E11 codes, they had insulin refills and they had at most three of OAD refill over 180 days of the first insulin refill, but at least 6 of insulin refills

3. (without ICD): they had no E10 nor E11 code, they had insulin refills and had at most three of OAD refills over 180 days of the first insulin refill, but at least 6 of insulin refills, age was <=35 at first refill

4. (some OAD combination): they had E10 code, the number of E10 codes were more or equal to the number of E11 codes, their first refill was insulin and there were only insulin refills during the first 180 days; then there were OAD refills with an insulin refill in the 7 days neighborhood of each

5. (some OAD): they had E10 code, the number of E10 codes were more or equal to the number of E11 codes, their first refill was insulin and there were only insulin refills during the first 180 days, and further OAD refills without an insulin refill in the 7 days neighborhood of each, but the last refill was insulin again

6. (some OAD and combination): they had E10 code; the number of E10 codes were more or equal to the number of E11 codes, their first refill was insulin and there were only insulin refills during the first 180 days, further, there were OAD refills with or without an insulin refill in the 7 days neighborhood of each, but the last refill was insulin or OAD+insulin in 7 days again

We considered patients having T2DM in case they did not meet the above criteria or were at least 40 years old at diagnosis.

Adapted from *Abonyi-Tóth Z, Rokszin G, Fábián I, et al. Incident Cancer Risk in Patients with Incident Type 2 Diabetes Mellitus in Hungary (Part 1). Cancers (Basel). 2024;16(9):1745. Published 2024 Apr 29. doi:10.3390/cancers16091745* upon a Creative Commons CC BY licence.
